# Chromosomal Anomalies in Fetuses With Increased Nuchal Translucency: A Vietnamese Retrospective Study

**DOI:** 10.7759/cureus.72235

**Published:** 2024-10-23

**Authors:** Tuan M Vo, Ngoc T Hoang, Toan T Nguyen, Hoang Tran, Huong N Trinh

**Affiliations:** 1 Obstetrics and Gynecology, University of Medicine and Pharmacy at Ho Chi Minh City, Ho Chi Minh City, VNM; 2 Diagnostic Radiology, University of Medicine and Pharmacy at Ho Chi Minh City, Ho Chi Minh City, VNM; 3 Prenatal Care Unit, Tu Du Hospital, Ho Chi Minh City, VNM

**Keywords:** increased nuchal translucency (nt), live fetus, microarray, opy number of variants (cnv), pregnancy counseling

## Abstract

Background

Previously, fetuses with increased nuchal translucency (NT) were mainly tested for aneuploidy. Recent evidence has shown an incidence of other genetic disorders in euploidy fetuses with thickened NT. Chromosomal microarray analysis (CMA) and next-generation sequencing (NGS) can detect the incremental yield of microdeletions and microduplications (copy number variants (CNVs)) and provide useful information for prenatal counseling. This study aims to determine the frequency of pathogenic CNV (pCNV) and its associated factors in euploidy fetuses with increased NT.

Methods

This was a retrospective study of 491 fetuses with NT ≥ 3 mm that underwent chorionic villus sampling or amniocentesis and was tested either by CMA or CNV-seq at Tu Du Hospital between August 2020 and January 2022.

Results

A total of 491 cases with NT ≥ 3 mm were indicated for genetic testing. Among 397 euploidy fetuses, 36 (9.1%) were pCNV, 24 (6.0%) were submicroscopic pCNV (not visible by karyotyping), and 25 (6.3%) were variants of unknown significance (VUS). The most common pCNV were 22q11 duplication and 16p11.2-p12.2 deletion. The incidence of pCNV in fetuses with increased NT and other structural abnormalities was significantly higher than in fetuses with isolated increased NT in the first trimester (OR 3.75, 95% CI 1.79-7.86, p < 0.001). Maternal age and the thickness of NT were not associated with an increased risk of harboring pCNV.

Conclusion

CMA or CNV-seq can detect the incremental yield of pCNV in euploidy fetuses with increased NT to assist in more accurate prenatal counselling.

## Introduction

The measurement of nuchal translucency (NT) is a routine investigation performed during the first trimester of pregnancy. A thickened NT is associated with an increased risk of aneuploidy and structural abnormalities, especially congenital heart diseases [1-3]. However, a normal karyotype could still result in adverse outcomes in fetuses with increased NT. Previous literature has shown that the chance of live birth with no defects in the group with NT of 3.5-4.4 mm was 86%, and for those with NT of ≥ 6.5 mm, it was only 31% [4]. In these cases, structural anomalies may have been detected by late ultrasound or postnatally, thus complicating parental counseling.

High-resolution genetic testing, such as chromosomal microarray analysis (CMA) and next-generation sequencing (CNV-seq), has demonstrated that fetuses with increased NT might carry either aneuploidy, microdeletion, or microduplication, known as copy number variants (CNV) [5]. These chromosomal abnormalities may be associated with adverse pregnancy outcomes [6,7].

Currently, few studies report CNVs in fetuses with increased NT in Vietnam. This study aims to determine the incidence of pathogenic CNV (pCNV) and its association with clinical features in fetuses with increased NT.

## Materials and methods

At the Prenatal Care Department of Tu Du Hospital, pregnancies with NT ≥ 3 mm were subjected to first-trimester detailed ultrasound followed by counseling for CVS or amniocentesis. Genetic testing was either CMA or CNV-seq for aneuploidy and CNV. Additional imaging investigations included early detailed fetal ultrasound at 14 to < 20 weeks, fetal echocardiography, and detailed fetal ultrasound at 20-24 weeks.

CMA in this study was performed at Tu Du Genetics Department, using the G5955A SurePrint G3 Human CGH ISCA v2 Microarray Kit, 8x60K (Agilent Technologies, Santa Clara, CA) with 60-mer oligonucleotide probes, with an average probe distance of 30 Kb. CNV-seq was performed using the NEBNext kit 75 (New England Biolabs, Ipswich, MA) to cleave DNA. The DNA was sequenced using next-generation sequencing (NGS) technology on the NextSeq system (Illumina, San Diego, CA). Sequencing results were compared with the reference genome (hg19), which was subdivided into 100 Kb regions and used to identify the number of chromosomes and CNVs. CNV-seq only detects CNVs > 400 Kb.

CNVs were classified according to the American College of Medical Genetics (ACMG) guidelines 2020 [8]. pCNVs included those that were classified as “pathogenic” or “likely pathogenic.” A pregnancy with multiple CNV segments is considered pathogenic if ≥ 1 pCNV is present. Benign CNV includes cases classified as “benign” or “likely benign.” Pregnancies with multiple CNV segments, including variants of unknown significance (VUS) and benign, are classified as VUS. CNVs with a size ≥ 5 Mb were considered visible by the karyotype.

Gestational age was calculated according to crown rump length (CRL) at the time of NT ultrasound. Termination of pregnancy (TOP) was recorded as occurring at counseling after genetic results were available.

Data were processed using Stata 14.0 software (StataCorp LLC, College Station, TX). Continuous variables are expressed as mean ± standard deviation, while discrete variables are expressed as percentages. The correlation of variables and pCNV was determined by logistic regression (univariate, then synthesized by multivariate analysis to remove noise). Statistical significance was determined as p < 0.05.

## Results

In all 491 cases, the median maternal age was 31.2 ± 5.5 years old. The median gestational age at NT measurement was 12 weeks and three days, and the median NT thickness was 4.3 ± 1.2 mm (Table 1).

**Table 1:** Baseline characteristics of participants (n = 491) CMA: chromosomal microarray analysis; CNV: copy number variants; NT: nuchal translucency

Characteristics	n (%)
Mother age	
<25	54 (11%)
25-34	305 (62.1%)
≥35	132 (26.9%)
History of genetic abnormalities	
No	479 (97.6%)
Father/mother	2(0.4%)
Previous pregnancy	10 (2.0%)
Other ultrasound abnormalities	
No	287 (58.5%)
At NT scan	39 (7.9%)
Early detailed ultrasound (14 to <20 weeks)	102 (20.8%)
Detailed ultrasound (20-24 weeks)	42 (8.6%)
After detailed ultrasound	21 (4.3%)
Invasive procedure	
Chorionic villus sampling	180 (36.7%)
Amniocentesis	311(63.3%)
Genetic testing	
CMA	334 (68%)
CNV-seq	157 (32%)

**Figure 1:**
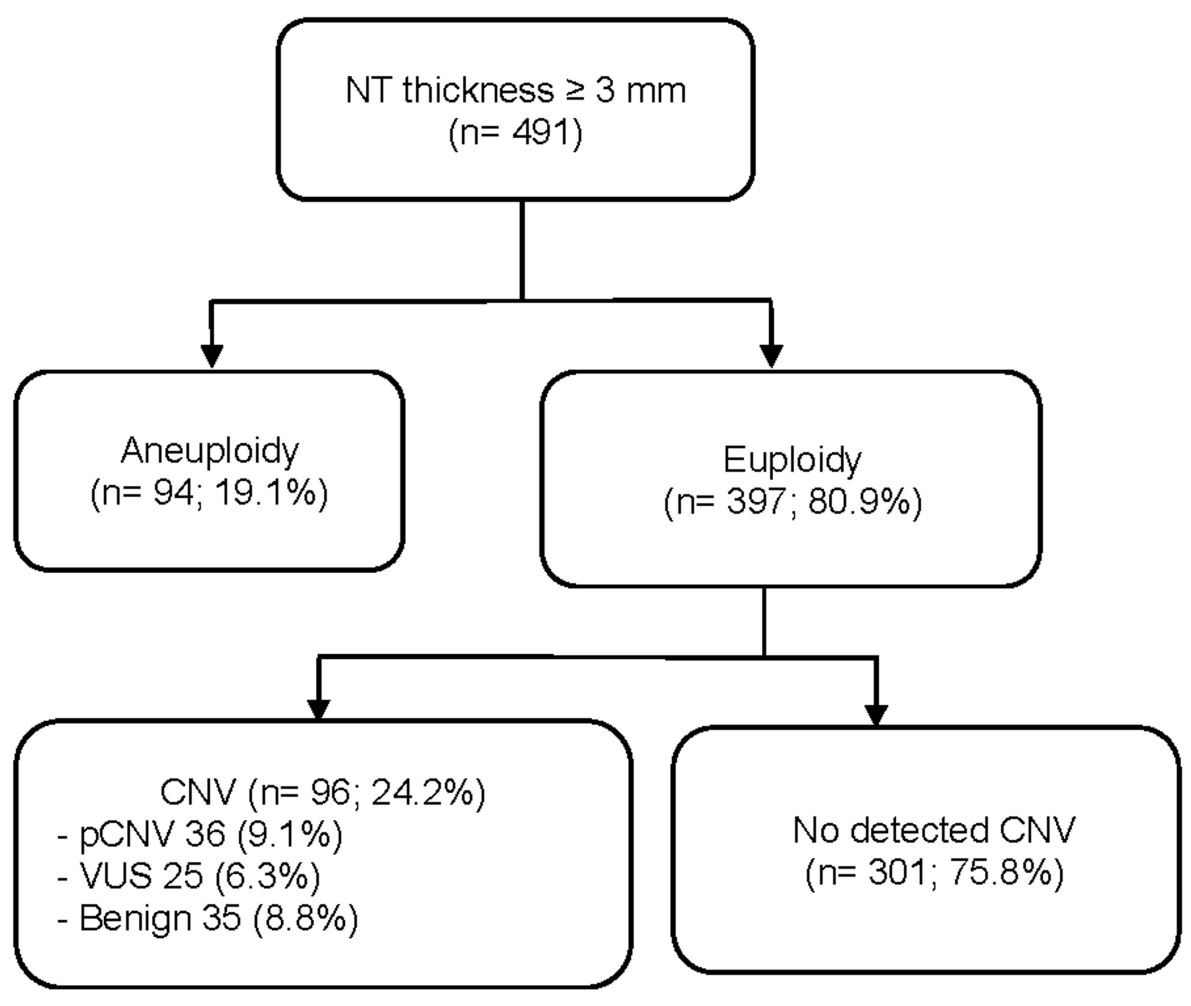
Flowchart of NT ≥ 3 mm and CMA or CNV-seq testing CMA: chromosomal microarray analysis; NT: nuchal translucency; pCNV: pathogenic copy number variants; VUS: variants of unknown significance

Through using CMA or CNV-seq testing to detect aneuploidy and CNV, there were 94/491 (19.1%) cases of aneuploidy and 397/491 (80.9%) cases of euploidy chromosomes. Of the 397 euploidy cases, 36 (9.1%) of these cases were classified as pCNV, among which 24 (6.0%) cases were considered sub-microscopic. The incidence of VUS was 6.3% (Figure 1). The most common chromosome carrying pCNV was chromosome 16 (14.3%) and chromosome 2 (11.9%). It was on each chromosome 10, 14, and 22 that 7.14% of cases detected pCNV. Only 188/491 (38.3%) cases were followed up until delivery or TOP at Tu Du Hospital, with the remaining cases being followed up at other hospitals. 

The thicker the NT, the higher the proportion of chromosomal disorders detected by CMA. However, there was no association with individual chromosomal abnormalities. The 5.5-6.4 mm NT group had the highest aneuploidy rate, while the ≥ 6.5 mm NT group had a higher proportion of pCNV and VUS than the other groups. The 3-3.4 mm and 5.5-6.4 mm NT groups had a lower proportion of pCNVs (Table 2).

**Table 2:** Distribution of chromosomal disorders by NT thickness Normal: no detecting CNV cases (n = 301) and benign CNV cases (n = 35). NT: nuchal translucency; pCNV: pathogenic copy number variants; VUS: variants of unknown significance

NT thickness	Normal	Aneuploidy	pCNV	VUS
3-3.4 mm (n = 116) (%)	82 (70.7%)	22 (19.0%)	5 (4.3%)	7 (6.0%)
3.5-4.4 mm (n = 206) (%)	137 (66.5%)	41 (19.9%)	20 (9.7%)	8 (3.9%)
4.5-5.4 mm (n = 96) (%)	70 (72.9%)	17 (17.7%)	6 (6.3%)	3 (3.1%)
5.5-6.4 mm (n = 49) (%)	34 (69.4%)	11 (22.5%)	2 (4.1%)	2 (4.1%)
≥6.5 mm (n = 24) (%)	13 (54.2%)	3 (12.5%)	3 (12.5%)	5 (20.8%)

Of the 397 cases of euploidy with increased NT, the median maternal age was 29.0 ± 4.9 years old, and the median NT was 4.3 ± 1.1 mm. These cases were divided into two groups: isolated increased NT group (group A) and increased NT with other malformations group (group B) found on NT ultrasound. There were 376 cases in group A, in which the percentage of pCNV, sub-microscopic pCNV, and VUS were 8.5%, 5.6%, and 6.4%, respectively, compared to these numbers for 21 cases in group B, which were 19.0%, 9.5%, and 4.8%, respectively (Table 3).

**Table 3:** CNV in pregnancy with increased NT Isolated increased NT or with other ultrasound abnormalities at NT scan. CNV: copy number variants; NT: nuchal translucency; pCNV: pathogenic copy number variants; VUS: variants of unknown significance

CNV	Isolated increased NT (n= 376) (%)	Other ultrasound abnormalities (n = 21) (%)	Total (n = 397)
No CNV	286 (76.1%)	15 (71.4%)	301 (75.8%)
pCNV	32 (8.5%)	4 (19%)	36 (9.1%)
Submicroscopic pCNV	22 (5.6%)	2 (9.5%)	24 (6.0%)
VUS	24 (6.4%)	1 (4.8%)	25 (6.3%)
Benign	34 (9.0%)	1 (4.8%)	35 (8.8%)

Analysis of the association with pCNV. To find factors associated with pCNV, we initially performed univariate analysis for 10 pairs of variables. In the second step, we selected five pairs of univariate analysis with a p-value of p < 0.25 into the multivariate analysis to mitigate against confounding factors. In the univariate analysis, the association between the NT 3-3.4 mm and the NT ≥ 4.5 mm group, with the risk of pCNV, showed no association (p = 0.609). Therefore, it was not included in the multivariate analysis.

The clinical significance of VUS is not clear, and benign CNV was usually considered a population polymorphism. Therefore, the VUS cases were not included in the association analysis, and samples with benign CNV were classified as normal in this study. When included in multivariate analysis, maternal age was not associated with pCNV. Increased NT with other ultrasound abnormalities was associated with an increased risk of carrying pCNV (OR 3.83, 95% CI 1.79-7.86, p < 0.001) (Table 4).

**Table 4:** Factors associated with pCNV OR: univariate logistic. OR*: multiple variate logistic. P*: multiple variate logistic. (-) reference group. Normal group includes cases with no detected CNV and benign CNV. pCNV: pathogenic copy number variants

Characteristics	pCNV (n = 36) (%)	Normal (n = 336) (%)	OR (95% CI)	OR* (95% CI)	P*
Mother age					
<25	7 (17.5%)	37 (11.0%)	1	1	-
25-34	28 (70.0%)	218 (64.9%)	0.71 (0.27-1.84)	0.77 (0.27-2.21)	0.625
≥35	5 (12.5%)	81 (24.1%)	0.38 (0.11-1.33)	0.55 (0.14-2.15)	0.386
Occupation					
Labor worker	11 (30.6%)	154 (45.8%)	1	1	-
Office worker	14 (38.8%)	117 (34.8%)	1.68 (0.73-0.98)	1.51 (0.62-3.67)	0.362
Housewife	11 (30.6%)	65 (19.4%)	2.37 (0.98-5.74)	2.41 (0.96-6.06)	0.060
History of pregnancy					
Nulliparous	18 (50.0%)	110 (32.7%)	1	1	-
Multiparous	18 (50.0%)	226 (67.3%)	0.49 (0.24-0.97)	0.52 (0.24-1.11)	0.091
Maternal BMI before pregnancy					
Underweight	2 (5.6%)	23 (6.8%)	1	1	-
Normal	23 (63.9%)	219 (65.2%)	1.21 (0.27-5.45)	1.33 (0.28-6.34)	0.721
Overweight	11 (30.6%)	94 (28.0%)	1.35 (0.28-6.49)	1.40 (0.27-7.15)	0.686
Other ultrasound abnormalities					
No	12 (33.3%)	221 (65.8%)	1	1	-
Yes	24 (66.7%)	16 (4.8%)	3.84 (1.85-7.96)	3.75 (1.79-7.86)	0.000

At the time of genetic results counseling, the autosomal aneuploidy cases largely chose TOP, accounting for 78/81 cases (96.3%). Meanwhile, due to aneuploidy of sex chromosomes, most families chose to follow up on pregnancy in 12/13 (92.3%) cases. A case of 45,X with ventricular septal defect, misplaced aorta was terminated of pregnancy.

The pCNV group had 11/36 cases (30.6%), which opted for TOP. The VUS group had a lower rate of TOP at 3/25, accounting for 12% of VUS cases. Two of these cases did not show malformations on ultrasound, and one case existed with increased nuchal fold. After having normal CMA or CNV-seq results, there were only 1/336 cases where TOP was performed (due to fetal hydrops, clubfoot) and four cases of intrauterine demise (Table 5).

**Table 5:** Management post-genetic testing pCNV: pathogenic copy number variants; TOP: termination of pregnancy (at the time of genetic results counseling); VUS: variants of unknown significance

Genetic testing	Miscarriage	TOP	Follow-up
Aneuploidy (n = 94) (%)	1 (1.1%)	77 (81.9%)	16 (17.2%)
Autosomal aneuploidy (n = 81) (%)	1(1.2%)	78 (96.3%)	2 (2.5%)
Sex chromosomal aneuploidy (n = 13) (%)	0	1 (7.7%)	12 (92.3%)
pCNV (n = 36) (%)	0	11 (30.6%)	25 (69.4%)
VUS (n = 25) (%)	0	3 (12%)	22 (88%)
Normal (n = 336) (%)	4 (1.2%)	1 (0.3%)	331 (98.5%)

## Discussion

Although increased NT may be an isolated factor, it may also be an early indicator of genetic disorders. Increased NT has been shown to be strongly associated with aneuploidy and structural abnormalities. Recently, it was found to be associated with CNV, or even single gene disorders causing adverse pregnancy outcomes [5,9].

Ultrasound in the first trimester can detect increased NT, which can cause anxiety for mothers and families. Counseling in cases of increased NT is common for obstetricians, fetal medicine practitioners, and genetic physicians. However, the current information given is largely based on data published abroad. There have been limited studies on new findings on high-resolution genetics detectable microdeletions and microduplications in fetuses with increased NT in Vietnam. The present study was carried out with the aim of providing data for parental counseling that is more representative of the demographic in Vietnam for use in clinical practice.

In this study of 491 cases of NT ≥ 3 mm, 19.1% were cases of aneuploidy. Among 397 euploidy fetuses, we found 9.1% were cases of pCNV, including 6% sub-microscopic pCNV. These results align with previous studies, as shown in Table 6. Comparison of the reported diagnostic rates of CNV in pregnancies with increased NT is challenging due to factors such as varying sample sizes, fetal characteristics, NT cut-offs, sub-microscopic CNV thresholds (varying from 5-10 Mb), and the resolution of CMA platforms. The results of CNV classifications have been continuously changed based on updated medical reports.

**Table 6:** Comparison of the incidence of pCNV with other studies (+) Other structural abnormalities; (-) Isolated increased NT; pCNV: pathogenic copy number variants; VUS: variants of unknown significance

Author	n	NT cut-off	Ultrasound abnormalities	Test	Submicroscopic pCNV (VUS) (%)	pCNV (VUS) (%)
Sinajon et al. (2020) [9]	107	3.5 mm	+	Array-CGH + SNP	2.8%	8.2 (4.6%)
Jin et al. (2021) [10]	87	3.0 mm	-	CNV-seq	5.7%	10.3 (1.1%)
Lund et al. (2015) [11]	94	3.5 mm	-	Array-CGH	8.2%	12.8 (3.2%)
Egloff et al. (2018) [12]	599	3.5 mm	-	Array-CGH	1.8%	2.7 (1.3%)
Our study	397	3.0 mm	+	Array-CGH + CNV-seq	6.0%	9.1 (6.3%)

In the present study, to standardize the classification of CNV, we followed the guidelines of ACMG 2020 [8] as used by Jin et al. and Lund et al. [10,11]. The results of our pCNV frequency are quite similar to those of the above two authors, and the results in the review and meta-analysis by Grande et al. are the same as others [5,10,11].

However, our VUS rate was higher, possibly due to a smaller sample size and different genetic testing resolution. In this study, up to 96 CNV were found, which may be the cause of the higher rate of VUS compared to other studies. In addition, we have not been able to do genetic testing for parents (Trio) in a uniform way to analyze VUS results based on a review of existing literatures [10-12].

In this study, the most common pCNVs were on chromosomes "16, 2, 22, 10, and 14." Jin et al. found that the most common pCNV-carrying chromosomes were chromosomes "22, 16, and 2" [10]. We found the most common pCNV was 22q11 duplication and 16p11.2-p12.2 microdeletion, which is consistent with the results of the above studies [5,10]. In all 36 cases of pCNV, there was no 22q11.2 deletion (DiGeorge syndrome) detected as previously reported. However, this result is consistent with the study of Egloff et al. (2018), where it was also noted that cases of sub-microscopic pCNV and strictly isolated increased NT may have excluded those harboring a 22q11.2 deletion [12]. The characteristics of pCNV cases are summarized in Table 7.

**Table 7:** Characteristics of pCNV cases CMA: chromosomal microarray analysis; CNV: copy number variants; NT: nuchal translucency; TOP: termination of pregnancy (at the time of genetic results counseling); VUS: variants of unknown significance; (-) none

CMA or CNV-seq result	Size (Kb)	NT ultrasound	Detailed ultrasound	TOP
Chr1:15982951_20215729del	4233	-	-	Yes
Chr2:25287175_25841368del	554	-	19.6w: pyelectasis	No
Chr2:17019_37217881del Chr6:206749_3189972del	37200 2983	Cystic hygroma, fetal hydrops	-	Yes
Chr2:110862477_111128847del	266	-	-	No
Chr2:143224966_145276260del	2051	-	16.5w: single umbilical artery; 32.4w: corpus callosum 5%	No
Chr2:145958505_156272325del Chr12:72719768_82483389del	10313 9764	-	35w: enlarged fetal heart, thickened ventricle wall, pericardial effusion	No
Chr4:123848799_124017270del	168	-	20w: IUGR, thickened NF, persistent left superior vena cava	No
Chr5:69238677_70771724dup	1533	-	29.1w: ventriculomegaly, echogenic bowel	No
Chr5:155600000_165500000dup	9900	-	14.1w: cystic hygroma	Yes
Chr9:204193_38815475dup Chr9:71069763_141006466dup	38611 69937	-	-	Yes
Chr10:136361_15903959del Chr17:75884558_81044553dup	10313 9764	-	14.6w: FL 2.5%, single umbilical artery	No
Chr10:73426703_79536149del	6109	-	-	No
Chr12:230421_13418897dup	13188	-	22.4w: thickened NF, Cerebellar 2%	No
Chr11:118940235_119478850dup	594	-	16.3w: hypoplastic nasal bone	No
Chr14:24577954_24981096dup	403	-	-	No
Chr14:29121637_29237889dup	116	-	20w: thickened NF	No
Chr14:34779294_35581683del	802	Cystic hygroma	16w: echogenic bowel, thickened nuchal fold; 20.5w: FL, HL <5%; 25.4w: enlarged fetal heart, thickened ventricle wall, pedal edema	No
Chr15:22765628-29030517dup	6265	Umbilical hernia	-	No
Chr15:83283503_84812693del	1529	-	19w: persistent left superior vena cava; 35w: IUGR	No
Chr16:6950959_7370706del	420	-	27w: thickened NF, FL < 5%	No
Chr16:29673954_30190568del	517	-	-	Yes
Chr16:21837492_22407931del	570	-	20.4w: thickened NF	No
Chr16:16900000_18100000del	1200	-	-	No
Chr16:87900000_90100000del	2200	-	-	Yes
Chr16:89079377_89388172del	309	-	-	No
Chr17:0_4300000del	4300	Umbilical hernia	-	Yes
Chr4:184717878_190896674dup Chr18:148963_14081887del	13932 6179	-	17.1w: cystic hygroma, thickened NF; 30.2w: FL <1%	No
Chr18:200000_3100000del	2900	-	18.6w: fifth finger clinodactyly	Yes
Chr19:6192888_7018231del	825	-	17.3w: echogenic bowel; 31.4w: MRI focal cortical dysplasia	Yes
Chr20:61632196_62244601del	612	-	20.3w: thickened NF	No
Chr11:4200000_5200000dup	1000	-	-	No
Chr22:17397498_51178264dup	33781	-	Thickened NF	Yes
Chr22:20700000_21500000dup	800	-	-	No
Chr22:19000000_21500000dup	2500	-	-	Yes
Chr19:10410426_53715810del (0,2)	43305	-	-	No
Chr19:637365_19960691del (0,3) Chr19:31023844_55748689del (0,2)	24724 19323	-	-	No

In addition, we found that maternal age was not associated with the risk of pregnancy with pCNV. This is consistent with other studies where increased maternal age was associated with aneuploidy risk but not with pCNV risk [9,13]. NT was thought to be related to the risk of aneuploidy, pCNV, and poor pregnancy outcomes in Lund et al. and Egloff et al. [11,12]. However, in our study, there was no statistically significant difference between NT thickness and risk of pCNV, as in Sinajon et al. (2020) and Jin et al. (2021) [9,10]. Furthermore, where structural abnormalities were found, we considered a significant association between additional accompanying structural abnormalities in increased NT fetuses and increased risk of harboring pCNV, as in Grande et al. [5].

In the present study, NT 3-3.4 mm had 4.3% pCNV (Table 2). This ratio is similar and sometimes higher when compared to pCNV results in NT ≥ 3.5 mm groups in existing studies [12]. Therefore, we proposed that CMA testing is still recommended for fetuses with NT ≥ 3 mm. In fetuses without aneuploidy and CNV, the rate of ultrasound abnormalities and congenital heart defects was at least 115/336 (34.2%) and 22/336 (6.5%), respectively. Cases of normal CMA results may still carry the potential for single gene disorders. Therefore, ongoing ultrasound monitoring throughout the whole pregnancy should be conducted for late-appearing structural abnormalities. This allows for repeat analysis of the CNV results or indicates extra genetic testing and patient consultation.

Most cases of autosomal aneuploidy chose to terminate the pregnancy (96.3%) due to the potential of severe structural abnormalities and neurodevelopmental disorders (Table 5). Meanwhile, in cases of sex chromosomal aneuploidy, if not accompanied by severe structural abnormalities, 92.3% of patients chose to continue the pregnancy. At least from our cohort and patient demographic, sex chromosomal aneuploidy is no longer an abnormality that indicates TOP, as potential disabilities tend to be less severe, and patients can be counseled on how best to prepare.

After counseling of the genetic results, 30.6% of pCNV cases resulted in TOP (Table 5). These cases had large CNV deletions, contained multiple genes, or corresponded with genetic syndromes. In 11 cases of pCNV at the time of pregnancy termination, there were five cases with abnormalities in the soft markers, one case with a severe abnormality (fetal hydrops), and the remaining five cases did not detect any other structural abnormalities. There was one case following CMA results with chr19 (arr(GRCh37) 19p13.3p13.2 (6192888_7018231) x1) size 825 Kb at a 17-week ultrasound only identified echogenic bowel. The patient chose to continue with the pregnancy; at 31 weeks, ventriculomegaly appeared, and MRI showed cortical dysplasia, at which point the patient chose to terminate the pregnancy at a late gestational age. The TOP rate in our study was lower than in existing studies [9,10]. However, because not all cases were followed up until the end of pregnancy, it was possible that some patients terminated the pregnancy at other clinics. The COVID-19 pandemic may also have caused patients to be followed up at different hospitals where abnormalities were detected. Therefore, for uniformity, we only considered management at the time of genetic results counseling in this study.

Counseling for pCNV cases is not simple and needs coordination by a multi-specialty team of fetal medicine physicians, pediatricians, radiologists, and geneticists. Analysis of CNV is based on many factors: CNV characteristics, related genetic syndromes, classification according to ACMG guidelines, comparison with cases carrying similar CNV reported on publications, the genetic history of the parents, and the phenotypic expression on ultrasound of the pregnancy. The purpose of counseling is not only to decide whether to terminate the pregnancy or not but also to orient the cause and prognosis, actively prepare and provide solutions for each problem that may be encountered after giving birth, in addition to counseling for future pregnancies.

Limitations of the study

The retrospective research method should only record available information. The patients were not followed up to the end of the pregnancy. It is not possible to fully describe ultrasound information and pregnancy outcomes at birth and after birth. In this study, we only stopped detecting CNV without having the conditions to study Trio or Exome sequencing to find the relationship between parental inheritance and single gene mutations in increased NT pregnancies.

## Conclusions

CMA should be performed in all pregnancies with NT ≥ 3 mm to identify incremental yield of pCNV. In our study, this rate was 9.1%. Cases not carrying CNV or VUS still have a risk of structural abnormalities appearing late. We need to continue the follow-up ultrasound investigation and after-birth manifestations. Further studies may be conducted with Exome sequencing for single-gene disorders in normal CMA cases.
